# Association between metabolic-associated fatty liver disease and risk of cardiometabolic multimorbidity: a disease trajectory analysis in UK Biobank

**DOI:** 10.3389/fendo.2025.1585725

**Published:** 2025-06-18

**Authors:** Yaqian Gao, Jianjun Yao, Suyi Liu, Song Yin, Zhenyu Jia, Yueqing Huang, Chunhua Zhao, Dingliu He

**Affiliations:** 1 Departments of General Medicine, The Affiliated Suzhou Hospital of Nanjing Medical University, Suzhou Municipal Hospital, Suzhou, Jiangsu, China; 2 Departments of General Medicine, The Huangqiao Subdistrict Community Health Service Center of Xiangcheng District, Suzhou, Jiangsu, China; 3 The First Clinical College, Anhui Medical University, Hefei, Anhui, China; 4 Department of General Medicine, Liuyuan Community Health Service Center, Suzhou, Jiangsu, China; 5 Department of General Medicine, The Affiliated Zhangjiagang Hospital of Soochow University, Suzhou, Jiangsu, China; 6 Department of Clinical Nutrition, Yancheng Clinical College of Xuzhou Medical University, First People’s Hospital of Yancheng, Yancheng, Jiangsu, China

**Keywords:** metabolic-associated fatty liver disease, cardiometabolic multimorbidity, disease trajectory, multistate model, UK Biobank

## Abstract

**Objective:**

While metabolic-associated fatty liver disease (MAFLD) has been associated with individual cardiometabolic diseases (CMDs), its role in the dynamic progression to cardiometabolic multimorbidity (CMM) remains unclear. We investigated the association of MAFLD, its severity and subtypes with CMM in individuals with no or one CMD at baseline.

**Methods:**

This prospective cohort study involved 386,651 individuals (344,415 without and 42,236 with a single CMD at baseline) from the UK Biobank. MAFLD was defined as the presence of hepatic steatosis plus overweight/obesity, type 2 diabetes (T2D), or metabolic abnormalities. CMM was defined as the coexistence of two or more CMDs in the same person, including T2D, coronary heart disease (CHD) and stroke. Cox proportional hazard models and multistate models were performed to estimate the hazard ratios (HRs) and 95% confidence intervals (95% CIs).

**Results:**

During a median follow-up of 13.85 years, 4,622 new-onset CMM cases emerged among participants free of CMD at baseline. MAFLD was significantly associated with an increased risk of incident CMM (adjusted HR: 2.78, 95% CI: 2.60-2.96). Multistate models showed that MAFLD adversely affected most transitions from baseline to single CMDs and then to CMM. Among the single-CMD participants, the adjusted HRs of incident CMM in the MAFLD group were 1.21 (95% CI: 1.13-1.31) for T2D patients, 1.90 (1.75-2.05) for CHD patients, and 1.65 (1.45-1.87) for stroke patients, respectively.

**Conclusion:**

MAFLD independently elevated the risk of incident CMM, regardless of the baseline CMD status. These findings emphasize the necessity of targeted MAFLD interventions for CMM prevention.

## 1 Introduction

Multimorbidity, commonly defined as the coexistence of two or more chronic diseases in the same person, is a prevalent phenomenon among middle-aged and older populations worldwide, along with rapid population ageing (1–4). The prevalence of multimorbidity is estimated to be 20-30% in the general population and rise to 55-98% in people aged 60 years and older (1). Due to the shared risk factors and similar pathobiological changes between cardiometabolic diseases (CMDs), cardiometabolic multimorbidity (CMM) was reported to be one of the replicable and harmful multimorbidity patterns (5–7). Generally, CMM was defined as the simultaneous presence of at least two of type 2 diabetes (T2D), coronary heart disease (CHD), and stroke (5). Mounting evidence demonstrated that CMM was significantly associated with a higher likelihood of premature mortality (5), depressive symptoms (8), cognitive impairment (9), and dementia (10). However, existing medical guides and treatments mainly focus on single CMDs and individuals with CMM are often excluded from the clinical trials (1, 7). Thus, deeply understanding the potential risk factors of CMM is substantially critical for the primary prevention of CMM and for alleviating the disease burden.

Non-alcoholic fatty liver disease (NAFLD) is the most common chronic liver disease worldwide and affects about a quarter of the global adult population, yet there are no effective treatments (11, 12). The existing diagnostic criteria of NAFLD include the presence of liver steatosis and the exclusion of excess alcohol consumption and other causes of liver disease (12). However, such an exclusive definition over-emphasizes the absence of alcohol drinking and neglects the cardiometabolic dysfunction related to heterogeneous disease characterization and adverse endpoints (13, 14). In 2020, an international panel of experts proposed an updated definition for NAFLD, named metabolic-associated fatty liver disease (MAFLD) (15). The new criteria are inclusive and emphasize the role of metabolic abnormalities in the incidence and development of liver diseases. Few studies have demonstrated that participants with MAFLD had a significantly increased risk of occurring individual CMDs, such as atrial fibrillation, myocardial infarction, ischemic stroke and heart failure, and even cancer (14, 16–21). However, the association of MAFLD with the subsequent risk of CMM has not yet been examined. Moreover, prior disease trajectory analyses of CMM pointed out that cardiometabolic risk factors disproportionally impacted the whole disease progression to CMM (22–24). To our knowledge, no prior studies have investigated whether and to which extent MAFLD impacts different transitions from the healthy status to single CMDs, and subsequently to CMM, which may provide crucial evidence to develop and implement targeted interventions for the onset and progression of CMM. We hypothesized that MAFLD could affect the whole trajectories of CMM development.

To address above knowledge gaps, we used a two-stage analytic strategy to investigate the association between MAFLD and the risk of incident CMM based on a prospective cohort study in the UK Biobank: (1) estimate the effect of MAFLD on transitions from a relatively healthy status (free of any CMD) to single CMDs (i.e., T2D, CHD and stroke) and to CMM in individuals with no CMD at baseline (stage one); (2) estimate the risk of developing CMM associated with MAFLD in individuals with a single CMD at baseline (stage two).

## 2 Materials and methods

### 2.1 Study population

The UK Biobank study is a large-scale prospective cohort study, and detailed information on its design and methods has been described previously (25). In brief, UK Biobank recruited more than 500,000 community-dwelling participants aged 37–73 years at 22 assessment centers across England, Wales, and Scotland between 2006 and 2010. Comprehensive information on sociodemographic characteristics, early-life experiences, lifestyle behaviors, health-related status and medication were collected via questionnaires, physical measurements, and biological sample assessment. The UK Biobank study was approved by the North West Multicenter Research Ethical Committee, and all participants provided written informed consent. This research was conducted under UK Biobank application number 104283.

**Figure 1:**
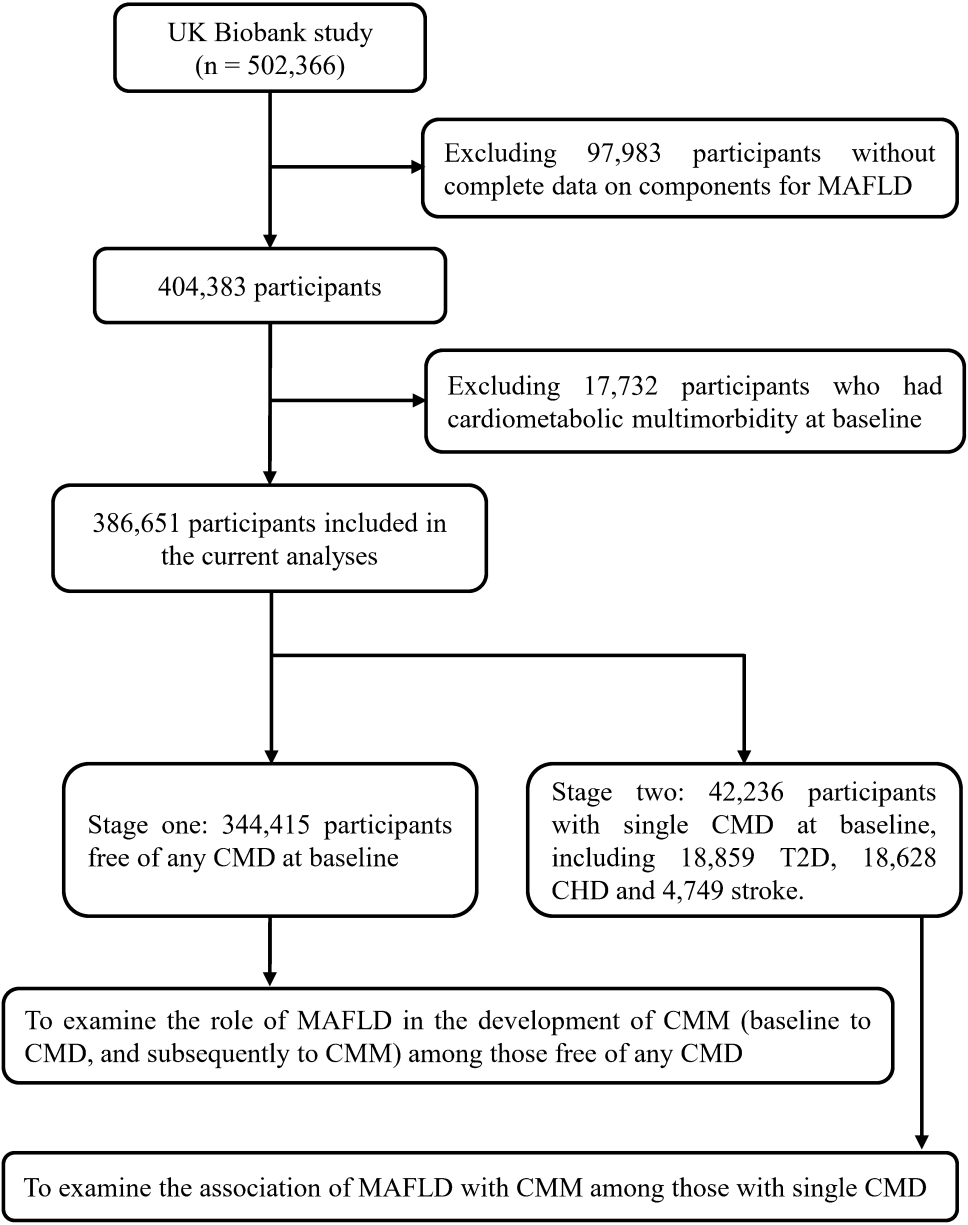
Flowchart of selecting study population. MAFLD, metabolic-associated fatty liver disease; T2D, type 2 diabetes; CHD, coronary heart disease; CMD, cardiometabolic disease; CMM, cardiometabolic multimorbidity.

**Figure 1** presented the flowchart of participants selection process. Of 502,366 participants in the UK Biobank, we excluded those without complete data on MAFLD components (n=97,983) and those with a diagnosis of CMM before the baseline survey (n=17,732). Finally, 386,651 participants, including 344,415 participants free of any single CMD at baseline and 42,236 participants with a single CMD at baseline, were included to estimate the association of MAFLD with the risk of incident CMM.

### 2.2 Definition of MAFLD

According to international expert consensus and past studies based on UKB (15, 19, 26, 27), MAFLD was defined as the presence of hepatic steatosis in addition to one of the following three criteria: (1) overweight or obesity (overweight: body mass index [BMI] ≥25 kg/m^2^; obesity: BMI ≥30 kg/m^2^), (2) presence of T2D, or (3) two or more metabolic abnormalities including prediabetes (hemoglobin A1c [HbA1c] ≥39 mmol/mol), lower high-density lipoprotein (HDL) cholesterol (<1.03 mmol/L for men, <1.29 mmol/L for women), hypertriglyceridemia (≥1.7 mmol/L or taking lipid-lowering medication), hypertension (systolic/diastolic blood pressure [SBP/DBP] ≥130/85 mmHg or taking antihypertensive medication), subclinical inflammation (high-sensitivity C-reactive protein >2 mg/L), and increased waist circumference (WC, ≥102 cm for men, ≥88 cm for women). Due to the absence of liver imaging and histological data in the UK Biobank, the fatty liver index (FLI) (28) was calculated using WC, gamma-glutamyl transferase, triglycerides, and BMI to define the hepatic steatosis (FLI ≥60), which had been widely used in prior UKB studies (19, 26, 27). The FLI had been verified to diagnose hepatic steatosis, with sensitivity and sensitivity of 87% and 86% (28). Notably, insulin resistance was not included in assessing metabolic abnormality due to the data availability in UK Biobank. The diagnosis of T2D was determined if participants met any of the following criteria: 1) had a self-reported diagnosis of T2D; 2) HbA1c > 47 mmol/mol; 3) took antidiabetic medication; 4) had International Classification Disease, version 10 (ICD-10) of E11 before the baseline assessment.

The severity of MAFLD was assessed via NAFLD fibrosis score calculated using the formula: −1.675+[0.037×age (years)]+[0.094×BMI (kg/m^2^)]+[1.13×T2D (yes=1, no=0)]+[0.99×AST/ALT ratio]-[0.013×platelet count(10^9^/L)]-[0.66×albumin(g/dL)] (29, 30). Participants were categorized into three groups according to this score: low (< -1.455), mild (-1.455 to 0.676), and severe (>0.676) liver fibrosis. Moreover, to assess the impact of different MAFLD subtypes, we further classified MAFLD into three subtypes: obese MAFLD (hepatic steatosis with only obesity), lean MAFLD (hepatic steatosis with only metabolic dysfunction), and obese and metabolic dysfunctional MAFLD (hepatic steatosis with obesity plus metabolic dysfunction). Because all individuals in stage one were free of T2D, we did not consider T2D in the MAFLD subtype.

### 2.3 Assessment of outcomes

The primary outcome was the incidence of cardiometabolic multimorbidity, which was defined as the simultaneous presence of two or three CMDs, including T2D, CHD and stroke (22, 23). In the UK Biobank study, disease incidence and diagnosis date were identified from the “first occurrence fields (Category ID: 1712)” based on the International Classification of Disease (ICD-10), which integrated information on self-reported disease history and linkage with electronic medical records and death registry records. Detailed information can be found at https://biobank.ndph.ox.ac.uk/showcase/label.cgi?id=1712. According to previous UKB studies on CMM (31–33), the corresponding ICD-10 codes were E11 for T2D, I20-I25 for CHD, and I60-I64 and I69 for stroke, respectively. For participants free of any CMD at baseline, the time of incident CMM was defined as the diagnosis date of occurring second CMD. For those with a single CMD at baseline, the time of incident CMM was the diagnosis date of another CMD.

### 2.4 Covariates

The potential covariates, including age, sex, ethnicity, educational levels, socioeconomic status, family income, employed status, lifestyle behaviors (smoking status, alcohol drinking, physical activity, sleep duration and diet), and family history of chronic disease, were collected through a self-reported questionnaire at baseline assessment. The Townsend deprivation index (TDI) is a measure of area-based socioeconomic status and derived from the residence postcode. Smoking and alcoholic drinking status were classified into never, previous, or current smoking/drinking. The levels of physical activity were estimated using the International Physical Activity Questionnaire short form, covering moderate and vigorous activities and walking over the last week. The total metabolic equivalent per week (METs, min/week) was then calculated to estimate the total volume of physical activity and categorized into three groups according to the tertiles. According to a previous study in UK Biobank (34), a healthy diet score was conducted by summing the following dietary factors: vegetables and fruits, fish, unprocessed red meat, and processed meat, and a higher score indicates a healthier diet. Sleeping duration was categorized into <7 h/day, 7–8 h/day, and >8 h/day, respectively. Family history of chronic disease included the occurrence of hypertension, diabetes, heart disease, and stroke in the participants’ father, mother and siblings.

### 2.5 Statistical analysis

Baseline characteristics were described as mean and standard deviation (SD) for continue variables and frequency (%) for categorical variables according to baseline MAFLD status. The group differences in baseline characteristics were compared using *t* test or *χ*^2^ test, as appropriate. For covariates with missing values or responses of “missing/unknown/prefer not to answer”, we created an additional category for categorical variables to maximize sample size and reduce the potential for inferential bias.

The Kaplan-Meier curves with a log-rank test were adopted to compare the cumulative incidence rate of CMM between the non-MAFLD and MAFLD groups. Cox proportional hazard models were conducted to estimate the hazard ratios (HRs) and 95% confidence intervals (95% CIs) for associations of MAFLD, its severity and subtypes with the risk of incident CMM. The proportional hazards assumption of Cox regression model was verified by the Schoenfeld residuals method, and results showed no significant deviation of assumption (all *P* values>0.05). Follow-up time was calculated from the date of baseline assessment to the date of incident CMM, death, or censoring (Nov 30, 2022), whichever came first. Three models were conducted: model 1 adjusted for age and sex; model 2 additionally adjusted for ethnicity, educational levels, family income, socioeconomic status, employed status, smoking status, alcohol drinking, physical activity, sleep duration and healthy diet score; model 3 additionally adjusted for family history of diabetes, hypertension, heart disease and stroke.

**Figure 2:**
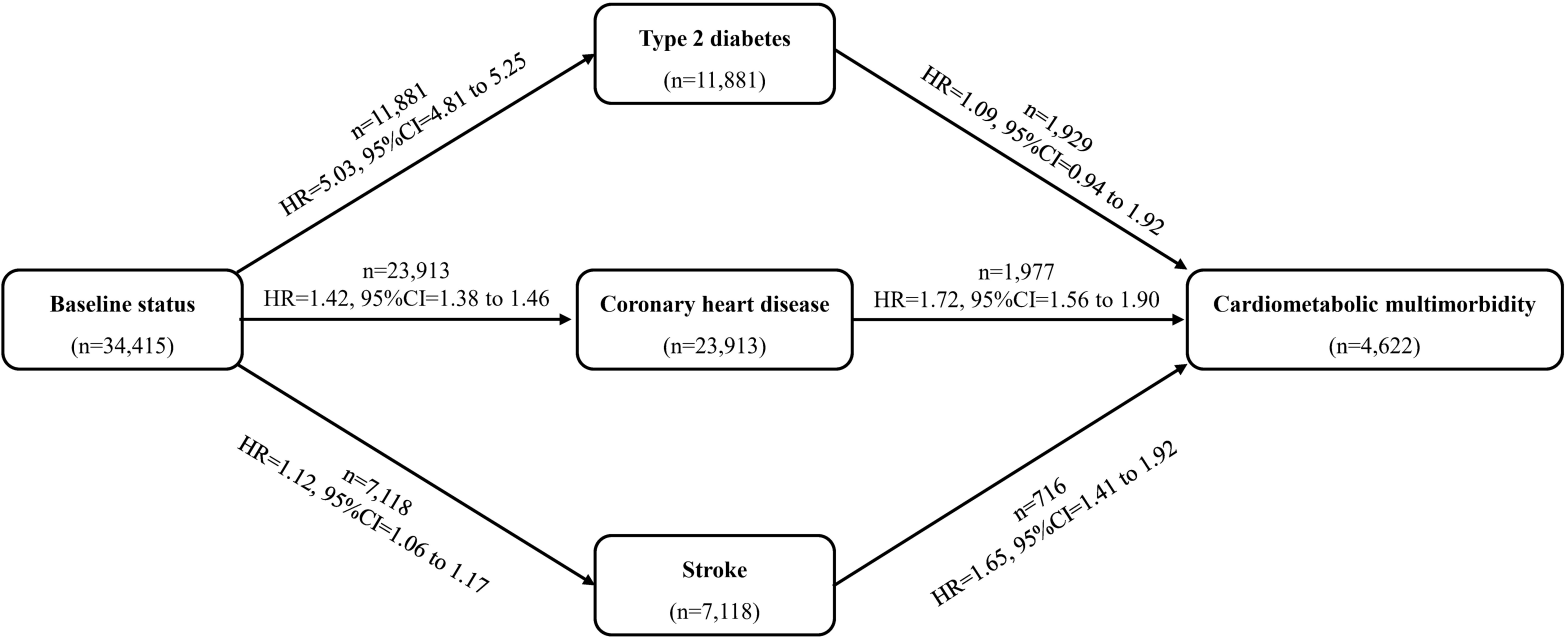
Role of metabolic-associated fatty liver disease (MAFLD) in the development of cardiometabolic multimorbidity. HR, hazard ratio; CI, confidence interval. Models adjusted for age, sex, ethnicity, educational levels, family income, socioeconomic status, employed status, smoking status, alcohol drinking, physical activity, sleep duration, healthy diet score, family history of diabetes, hypertension, heart disease and stroke.

Furthermore, we used the multi-state model to evaluate the role of MAFDL in the different transitions from baseline to single CMD and subsequently to CMM among individuals free of any CMD at baseline. As shown in **Figure 2**, we predefined a disease-developing framework with 6 transitions: 1) baseline status (free of any CMD) to T2D, 2) baseline status to CHD, 3) baseline status to stroke, 4) T2D to CMM, 5) T2D to CMM, and 6) stroke to CMM. The multi-state models were assumed to follow a Markov process, whereby the future state depends only on the current state and not on the prior history. This assumption is commonly applied in multistate models of disease progression and was deemed appropriate based on the structure and objectives of our analysis. The multi-state models were performed using the R statistic software “*mstate*” package (35).

Subgroup analyses were performed by age, sex, ethnicity, employment status, education level, socioeconomic status, current smoking, current drinking, physical activity, healthy diet score, sleep duration, and obesity. In addition, a series of sensitivity analyses were conducted to confirm the robustness of the results. First, we imputed missing covariate data using multivariate imputations by chained equations (MICE) based on random forest models. Second, to minimize the potential reverse causality, we conducted a landmark analysis that excluded new-onset cases of cardiometabolic multimorbidity occurring in the first two years of follow-up. Third, to account for the competing risk of mortality from other causes, we applied the Fine-Gray subdistribution hazard model to assess the associations by taking mortality as a competing event.

All statistical analyses were performed using the R software (version 4.3.3), and a two-sided *P*<0.005 represented statistical significance.

## 3 Results

### 3.1 Stage one: individuals free of any CMD at baseline

Of 344,415 participants free of any CMD in stage one, 223,852 participants were non-MAFLD (mean age: 55.73 ± 8.19 years and 66.31% female), and 120,563 participants had MAFLD (mean age: 56.55 ± 7.89 years and 37.08% female) at baseline. Compared with the non-MAFLD group, participants with MAFLD tended to be older, men, White, not employed, more deprived, less educated, and have a lower income (**Table 1**). They also had a higher prevalence of smoking, alcohol drinking, physical inactivity, inappropriate sleep duration, unhealthy diet, family history of heart disease, stroke, and diabetes, higher levels of BMI, WC, SBP, DBP, HbA1c, C reactive protein, triglycerides, and Gamma-glutamyltransferase, and lower HDL cholesterol (**Table 1**).

**Table 1:** Baseline characteristics of study population according to MAFLD.

Characteristic	Stage one individuals free of any CMD at baseline			Stage two individuals with single CMD at baseline		
	non-MAFLD group	MAFLD group	*P* value	non-MAFLD group	MAFLD group	*P* value
n	223852	120563		16716	25520	
Age, years	55.73 ± 8.19	56.55 ± 7.89	<0.001	60.45 ± 7.17	59.95 ± 6.95	<0.001
Gender			<0.001			<0.001
Female	148431 (66.31%)	44700 (37.08%)		8009 (47.91%)	8300 (32.52%)	
Male	75421 (33.69%)	75863 (62.92%)		8707 (52.09%)	17220 (67.48%)	
Ethnicity			<0.001			<0.001
White	203110 (90.73%)	110249 (91.45%)		14889 (89.07%)	23024 (90.22%)	
Non-white	20742 (9.27%)	10314 (8.55%)		1827 (10.93%)	2496 (9.78%)	
College degree			<0.001			<0.001
No	139799 (62.45%)	85509 (70.92%)		11814 (70.67%)	19510 (76.45%)	
Yes	81885 (36.58%)	33553 (27.83%)		4608 (27.57%)	5538 (21.70%)	
Missing	2168 (0.97%)	1501 (1.24%)		294 (1.76%)	472 (1.85%)	
Family income			<0.001			<0.001
<£18000	37172 (16.61%)	23973 (19.88%)		4602 (27.53%)	7792 (30.53%)	
£ 18000~51999	99790 (44.58%)	54206 (44.96%)		6697 (40.06%)	10372 (40.64%)	
>£ 51999	55017 (24.58%)	25946 (21.52%)		2309 (13.81%)	3134 (12.28%)	
Missing	31873 (14.24%)	16438 (13.63%)		3108 (18.59%)	4222 (16.54%)	
Employ status			<0.001			<0.001
Current employ	136039 (60.77%)	71405 (59.23%)		6322 (37.82%)	10135 (39.71%)	
Not employ	86804 (38.78%)	48553 (40.27%)		10285 (61.53%)	15233 (59.69%)	
Missing	1009 (0.45%)	605 (0.50%)		109 (0.65%)	152 (0.60%)	
TDI score	-1.55 ± 2.95	-1.21 ± 3.12	<0.001	-1.02 ± 3.25	-0.60 ± 3.35	<0.001
Smoking status			<0.001			<0.001
Never smoking	132375 (59.14%)	60135 (49.88%)		8316 (49.75%)	10437 (40.90%)	
Previous smoking	68743 (30.71%)	46050 (38.20%)		6313 (37.77%)	11909 (46.67%)	
Current smoking	21849 (9.76%)	13746 (11.40%)		1964 (11.75%)	2949 (11.56%)	
Missing	885 (0.40%)	632 (0.52%)		123 (0.74%)	225 (0.88%)	
Alcohol drinking			<0.001			0.1
Never drinking	8975 (4.01%)	4601 (3.82%)		1173 (7.02%)	1660 (6.50%)	
Previous drinking	6670 (2.98%)	4178 (3.47%)		961 (5.75%)	1540 (6.03%)	
Current drinking	207777 (92.82%)	111491 (92.48%)		14511 (86.81%)	22226 (87.09%)	
Missing	430 (0.19%)	293 (0.24%)		71 (0.42%)	94 (0.37%)	
Sleep duration			<0.001			<0.001
7–8 hours/day	157337 (70.29%)	77972 (64.67%)		10668 (63.82%)	14902 (58.39%)	
<7 hours/day	50828 (22.71%)	31987 (26.53%)		4249 (25.42%)	7176 (28.12%)	
>8 hours/day	14402 (6.43%)	9677 (8.03%)		1621 (9.70%)	3098 (12.14%)	
Missing	1285 (0.57%)	927 (0.77%)		178 (1.06%)	344 (1.35%)	
Physical activity			<0.001			<0.001
Q1	51179 (22.86%)	35896 (29.77%)		3877 (23.19%)	7963 (31.20%)	
Q2	61460 (27.46%)	29207 (24.23%)		4147 (24.81%)	5515 (21.61%)	
Q3	63643 (28.43%)	27323 (22.66%)		4443 (26.58%)	5188 (20.33%)	
Missing	47570 (21.25%)	28137 (23.34%)		4249 (25.42%)	6854 (26.86%)	
Healthy diet score	3.46 ± 1.28	3.05 ± 1.33	<0.001	3.47 ± 1.33	3.12 ± 1.35	<0.001
BMI, kg/m^2^	24.86 ± 2.83	31.18 ± 4.26	<0.001	25.49 ± 2.76	32.48 ± 4.99	<0.001
WC, cm	82.39 ± 8.87	101.60 ± 9.37	<0.001	86.47 ± 8.61	105.95 ± 11.08	<0.001
SBP, mmHg	134.81 ± 18.64	142.95 ± 17.49	<0.001	137.68 ± 18.70	141.78 ± 17.78	<0.001
DBP, mmHg	80.30 ± 9.74	86.46 ± 9.56	<0.001	78.94 ± 9.88	83.34 ± 10.00	<0.001
HDL cholesterol, mmol/L	1.58 ± 0.38	1.28 ± 0.29	<0.001	1.43 ± 0.37	1.18 ± 0.28	<0.001
HbA1c, mmol/mol	34.44 ± 3.52	35.74 ± 3.91	<0.001	40.74 ± 11.31	45.81 ± 13.64	<0.001
C reactive protein, mg/L	1.93 ± 3.78	3.55 ± 4.70	<0.001	2.27 ± 4.47	3.89 ± 5.36	<0.001
Triglycerides, mmol/L	1.34 ± 0.62	2.40 ± 1.17	<0.001	1.35 ± 0.62	2.36 ± 1.21	<0.001
Gamma-glutamyltransferase, U/L	26.02 ± 20.74	53.94 ± 54.94	<0.001	30.21 ± 26.44	59.91 ± 65.55	<0.001
Family history of disease						
Heart disease	91938 (41.07%)	51145 (42.42%)	<0.001	8708 (52.09%)	13216 (51.79%)	0.537
Stroke	57258 (25.58%)	31151 (25.84%)	0.096	5165 (30.90%)	7385 (28.94%)	<0.001
Diabetes	41670 (18.61%)	27629 (22.92%)	<0.001	4485 (26.83%)	8293 (32.50%)	<0.001
Hypertension	106914 (47.76%)	57084 (47.35%)	0.021	7781 (46.55%)	12336 (48.34%)	<0.001

MAFLD, metabolic-associated fatty liver disease; CMD, cardiometabolic disease; TDI, Townsend deprivation index; BMI, body mass index; WC, waist circumference; SBP, systolic blood pressure; DBP, diastolic blood pressure.

Analyses in stage one were conducted among individuals without any CMD at baseline; analyses in stage two were conducted among individuals with single CMD at baseline. Describe statistics were shown as mean ± standard deviation for continue variables and number (%) for categorized variables.

During a median follow-up period of 13.85 years, we identified 4,622 new-onset CMM cases (1,536 in the non-MAFLD group and 3,086 in the MAFLD group). The incidence rates were 0.50 per 10,00 person-years in the non-MAFLD group and 1.86 per 10,00 person-years in the MAFLD group, respectively. As shown in **Table 2**, **Supplementary Figure 1**, individuals with MAFLD had a significantly increased risk of incident CMM, and the risk was raised with the increment in MAFLD severity. After fully adjusting for potential covariates (Model 3), Cox proportional hazard models revealed that the risk of incident CMM was 2.78 times (HR: 2.78, 95% CI: 2.60-2.96) higher in the MAFDL group than the non-MAFLD group. In terms of different severity of MAFLD, the fully adjusted HRs of incident CMM were 2.63 (95% CI: 2.46-2.81) for the MAFLD with no fibrosis group, 3.73 (95% CI: 3.38-4.12) for the MAFLD with mild fibrosis group, and 4.00 (95% CI: 1.66-9.64) for the MAFLD with severe fibrosis group, respectively (**Table 2**). For different subtypes of MAFLD, participants in the lean MAFLD group or the obese and metabolic dysfunctional MAFDL group had 1.96 (HR: 1.96, 95% CI: 1.49-2.58) and 2.87 times (HR: 2.87, 95% CI: 2.69-3.07) elevated risk of developing CMM than those in non-MAFLD group, but the risk was not statistically significant in the obese MAFLD group (HR: 1.12, 95% CI: 0.87-1.45) (**Supplementary Table 1**).

**Table 2:** Association between metabolic-associated fatty liver disease (MAFLD) and cardiometabolic multimorbidity.

Exposure	n	Cases/person-years	Unadjusted	Model 1	Model 2	Model 3
Individuals free of cardiometabolic disease at baseline						
Non-MAFLD	223,852	1,536/3,092,787	Ref.	Ref.	Ref.	Ref.
MAFLD	120,563	3,086/1,657,654	3.76 (3.53-3.99)	3.19 (2.99-3.40)	2.86 (2.68-3.04)	2.78 (2.60-2.96)
MAFLD with low fibrosis	107,817	2,499/1485,362	3.39 (3.18-3.61)	3.00 (2.81-3.20)	2.70 (2.53-2.89)	2.63 (2.46-2.81)
MAFLD with mild fibrosis	12,644	582/170,898	6.97 (6.33-7.66)	4.42 (4.01-4.88)	3.85 (3.49-4.25)	3.73 (3.38-4.12)
MAFLD with severe fibrosis	102	5/1394	7.28 (3.03-17.51)	5.37 (2.23-12.92)	4.18 (1.74-10.08)	4.00 (1.66-9.64)
Individuals with type 2 diabetes at baseline						
Non-MAFLD	5,485	953/69,373	Ref.	Ref.	Ref.	Ref.
MAFLD	13,374	3,087/164,272	1.37 (1.28-1.47)	1.29 (1.20-1.39)	1.23 (1.14-1.32)	1.21 (1.13-1.31)
MAFLD with low fibrosis	4,821	964/60,444	1.16 (1.06-1.27)	1.25 (1.14-1.37)	1.19 (1.08-1.30)	1.17 (1.07-1.29)
MAFLD with mild fibrosis	8,015	1,953/97,590	1.46 (1.35-1.58)	1.29 (1.19-1.39)	1.23 (1.14-1.33)	1.21 (1.12-1.31)
MAFLD with severe fibrosis	538	170/6,237	2.00 (1.70-2.36)	1.69 (1.43-1.99)	1.58 (1.34-1.86)	1.55 (1.31-1.83)
Individuals with coronary heart disease at baseline						
Non-MAFLD	8,645	984/113,698	Ref.	Ref.	Ref.	Ref.
MAFLD	9,983	2,170/124,946	2.02 (1.88-2.18)	2.02 (1.88-2.19)	1.91 (1.77-2.07)	1.90 (1.75-2.05)
MAFLD with low fibrosis	7,720	1,583/97,507	1.89 (1.74-2.05)	1.91 (1.76-2.07)	1.81 (1.66-1.96)	1.79 (1.65-1.94)
MAFLD with mild fibrosis	2,234	574/27,156	2.48 (2.23-2.75)	2.39 (2.15-2.65)	2.26 (2.03-2.51)	2.23 (2.00-2.48)
MAFLD with severe fibrosis	29	13/283	5.56 (3.22-9.62)	5.46 (3.16-9.43)	5.25 (3.03-9.08)	5.32 (3.07-9.22)
Individuals with stroke at baseline						
Non-MAFLD	2,586	431/32,925	Ref.	Ref.	Ref.	Ref.
MAFLD	2,163	625/25,780	1.86 (1.65-2.10)	1.76 (1.56-2.00)	1.66 (1.46-1.88)	1.65 (1.45-1.87)
MAFLD with low fibrosis	1,857	509/22,376	1.75 (1.53-1.98)	1.69 (1.48-1.92)	1.58 (1.39-1.81)	1.57 (1.38-1.79)
MAFLD with mild fibrosis	304	116/3,377	2.64 (2.15-3.25)	2.26 (1.84-2.79)	2.15 (1.74-2.65)	2.16 (1.74-2.66)

MAFLD, metabolic-associated fatty liver disease.

Data were presented as hazard ratios (95% confidence intervals).

Model 1 adjusted for age and sex;

Model 2 adjusted for model 1 plus ethnicity, educational levels, family income, socioeconomic status, employed status, smoking status, alcohol drinking, physical activity, sleep duration and healthy diet score;

Model 3 adjusted for model 2 plus family history of diabetes, hypertension, heart disease and stroke.

**Figure 3:**
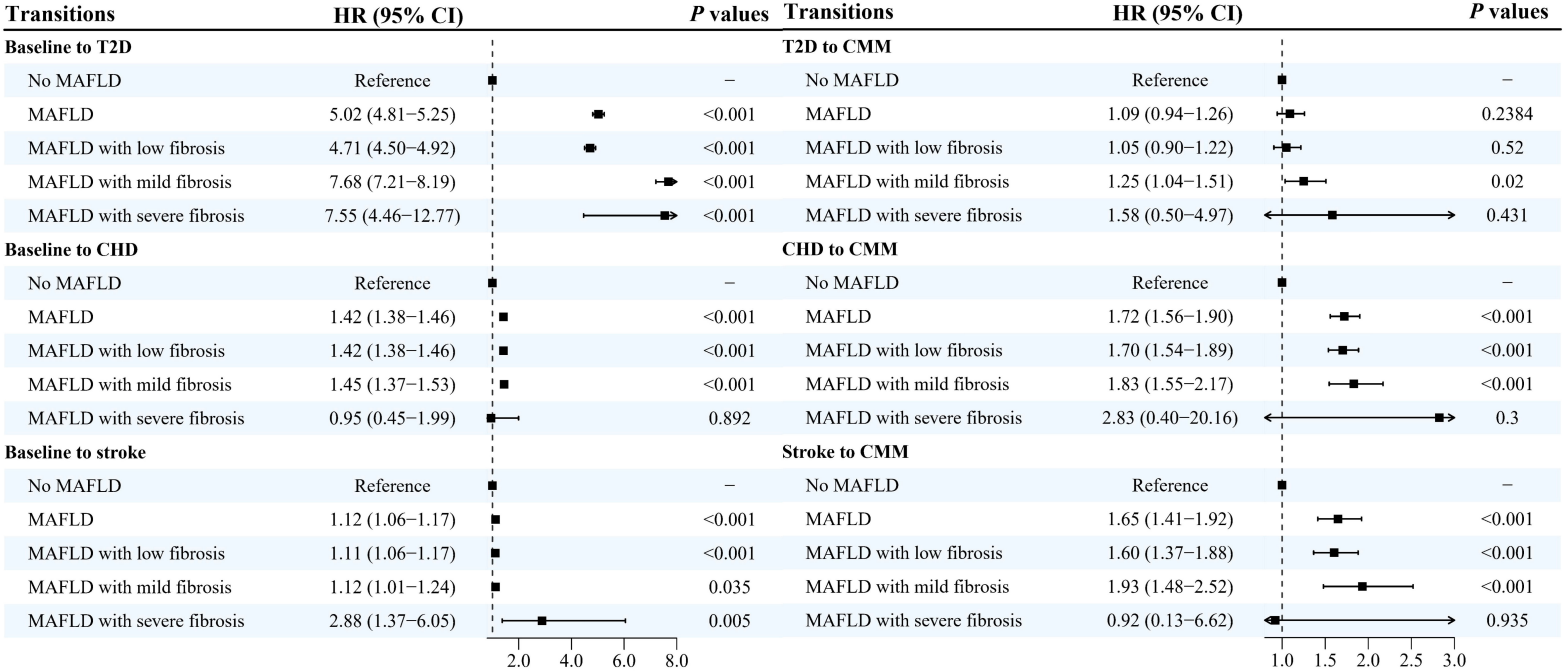
Hazard ratios and 95% confidence intervals for associations between severity of metabolic-associated fatty liver disease and the development of cardiometabolic multimorbidity HR, hazard ratio; CI, confidence intervals; MAFLD, metabolic-associated fatty liver disease; T2D, type 2 diabetes; CHD, coronary heart disease; CMM, cardiometabolic multimorbidity. Multi-state models were conducted to estimate the role of MAFLD in the development of cardiometabolic multimorbidity, and disease transitions were presented in **Figure 2**. Model adjusted for age, sex, ethnicity, educational levels, family income, socioeconomic status, employed status, smoking status, alcohol drinking, physical activity, sleep duration, healthy diet score, family history of diabetes, hypertension, heart disease and stroke.

Results from the multi-state models showed that MAFLD was positively and significantly associated with all transitions from baseline to CMM, except the transition from T2D to CMM (**Figures 2**, **3**, **Supplementary Figure 2**). After adjusting for potential covariates, compared with the non-MAFLD group, the fully adjusted HRs of MAFLD were 5.03 (95% CI: 4.81-5.25) for the transition from baseline to T2D, 1.42 (95% CI: 1.38-1.46) for the transition from baseline to CHD, 1.12 (95% CI: 1.06-1.17) for the transition from baseline to stroke, 1.09 (95% CI: 0.94-1.92) for the transition from T2D to CMM, 1.72 (95% CI: 1.56-1.90) for the transition from CHD to CMM, and 1.65 (95% CI: 1.41-1.92) for the transition from stroke to CMM, respectively. Moreover, as shown in **Figure 3**, **Supplementary Table 2**, different severities and subtypes of MAFLD showed various magnitudes for the associations with transitions from baseline to individual CMDs and subsequently to CMM.

### 3.2 Stage two: individuals with a single CMD at baseline

A total of 42,236 individuals with a single CMD at baseline (18,859 T2D, 18,628 CHD and 4,749 stroke) were included in stage two, and 25,520 had MAFLD (mean age: 59.95 ± 6.95 and 32.52% female). As shown in **Table 1**, participants with MAFLD were more likely to be younger, male, White, less educated, employed, more deprived, and have lower income than the non-MAFLD group. The prevalence of smoking, alcohol drinking, unrecommended sleep duration, physical inactivity, and unhealthy diet, the levels of BMI, WC, SBP, DBP, HbA1c, C reactive protein, triglycerides, and gamma-glutamyltransferase, lower level of HDL cholesterol were also observed in the MAFLD group (**Table 1**).

The Kaplan-Meier curves showed that the cumulative hazard of CMM was significantly higher in the MAFLD group than in the non-MAFLD group (all *P* values <0.001, **Supplementary Figure 1**). After full adjustment of covariates, compared with the non-MAFLD group, the HRs of incident CMM in the MAFLD group were 1.21 (95% CI: 1.13-1.31) for participants with T2D, 1.90 (1.75-2.05) for participants with CHD, and 1.65 (1.45-1.87) for participants with stroke, respectively (**Table 2**). Regard of different severity of MAFLD, the highest hazards of occurring CMM were found in the MAFLD with severe fibrosis group, with HRs of 1.55 (95% CI:1.31-1.83) in those with T2D, 5.32 (3.07-9.22) in those with CHD, and 2.16 (1.74-2.66) in those with stroke (in the mild fibrosis group). Among individuals with T2D at baseline, the subtypes of obese MAFLD and obese and metabolic dysfunction MAFLD had significant association with CMM (HR and 95% CI: 2.00, 1.10-3.63 and 1.22, 1.13-1.31), whereas the association was statistically significant in the obese and metabolic dysfunction MAFLD group among those with CHD (HR: 1.92, 95% CI: 1.78-2.08) or stroke (HR: 1.67, 95% CI: 1.47-1.90).

### 3.3 Additional analyses

Subgroup analyses showed that age (<60 *vs*. ≥60 years), gender (male *vs*. female) and current smoking status (no *vs*. yes) significantly modified the association of MAFLD with CMM risk among those with none CMD at baseline (all *P* values for interaction ≤0.001) (**Supplementary Figure 3**). The risk of incident CMM was significantly higher among those aged <60 years, females, and those who were not currently smoking than their corresponding counterparts. Among individuals with T2D at baseline, we found significant effect modification by current smoking status and physical activity, where increased risk of CMM was only significant in those who were not currently smoking and those with moderate or high physical activities (all *P* values for interaction <0.05, **Supplementary Figure 4**). Among individuals with CHD or stroke, the CMM risk was also more pronounced among those aged <60 years (all *P* values for interaction <0.05, **Supplementary Figures 5**, **6**). Results from sensitivity analyses persisted robustly after excluding those experiencing CMM within the first 2 years of follow-up (**Supplementary Table 3**), multiple imputation for missing data on covariates (**Supplementary Table 4**), and when considering the competing risk of mortality from other causes (**Supplementary Table 5**).

## 4 Discussion

In this prospective cohort study in the UK Biobank, we found that MAFLD significantly increased the future risk of incident CMM, regardless of the CMD status at baseline. Importantly, MAFLD had detrimental influences on all transitions from the baseline status (free of any CMD) to T2D, CHD and stroke, and then from CHD or stroke to CMM. Besides, we also identified the subpopulation susceptible to the higher risk of CMM conferred by MAFLD.

As a novel concept updated from NAFLD, the clinical significance of MAFLD has attracted much interest from researchers, and limited studies evaluated its associations with several intrahepatic and extrahepatic diseases (including cardiovascular diseases, CVDs) in different national studies (13, 16, 18–21). A nationwide cohort study of about 9 million middle-aged Koreans showed that MAFLD was associated with a 1.52 times elevated risk of occurring the composite CVD outcome and a 1.20-1.89 times higher risk of incident specific CVD subtypes or CVD-related mortality (16). Based on a retrospective study in the JMDC Claims Database, Ohno and colleagues also found positive associations of MAFLD with heart failure, atrial fibrillation, myocardial infarction, and stroke, and risks varied between MAFLD subtypes (20). Moreover, including 24,772 pairs of new-onset MAFLD cases and age- and sex-matched controls from the Kailuan Study, Zheng and colleagues investigated whether the association of MAFLD with CVD differed across the onset-age of MAFLD (21). Their results indicated that the CVD risk gradually declined with the increases in MAFLD onset age, and MAFLD cases younger than 45 years had the highest hazard, whereas the risk in those older than 55 years was not statistically significant (21). In line with previous investigations, our results provided additional evidence that MAFLD also witnessed a stronger association with CMM among individuals with no or one single CMD, and the hazards raised with the severity of MAFLD. These findings suggested that targeted and regular screening and monitoring should be developed and implemented to target not only individual CMDs but also CMM among people with MAFLD.

In terms of the developing progression of CMM, multistate models have been widely applied in prior studies to assess the impacts of lifestyle behaviors (22–24), depressive symptoms (31), handgrips strength (36), pulmonary function (37), and air pollution (33, 38). Most studies found the influences of specific factors on CMM progression varied across the disease stages. For instance, an investigation of the Whitehall II cohort study indicated that all lifestyle behaviors (e.g., physical activity, diet, alcohol consumption and smoking) and clinical profiles (e.g., hypertension, obesity and hyperlipidemia) were significantly related to the transition from disease-free status to the first CMD, but only smoking and obesity were associated with the transition from first CMD to CMM (23). Similarly, a study conducted in the China Kadoorie Biobank found that a composite lifestyle score consisting of heavy alcoholic drinking, tobacco smoking, poor diet, physical inactivity, and unhealthy body size had influences on each transition from healthy status to five CMDs, CMM and death, with a relatively greater risk in transitions to CMD than to CMM (22). Using the same data of the UK Biobank, previous researches have revealed the detrimental impacts of air pollutions (33, 38), depression symptoms (31), lower handgrip strength (36), low functional function (37), physical inactivity (39), frailty (40), and poor diet (32) on the different stages of CMM incidence, progress and prognosis. In the present study, our results further demonstrated that MAFLD affected the CMM progression and the influences depended on the disease stages and MAFLD subtypes. The results of multistate models were further confirmed in the analyses of participants with single CMDs at baseline (stage two). Differed from most previous studies using the first CMD stage in CMM progression (23, 32, 36, 40), we distinguished the individual CMDs into T2D, CHD and stroke, and conducted six disease transitions between healthy status, individual CMDs and CMM, which might provide evidence for the precise prevention of CMM and give an alternative analytic strategy for future studies. However, due to the relatively limited CMM cases and more complex disease transitions, we did not include death as an absorbing endpoint in our analyses. Future studies are warranted to investigate the association of MAFLD with cause-specific deaths.

Our subgroup analyses indicated that among people with no CMD at baseline, those younger than 60 years, females, and current non-smokers were more susceptible to the elevated risk of CMM incidence associated with MAFLD than their counterparts. Similarly, a greater risk was also observed in CHD and stroke cases younger than 60 years. Compared with the elderly, younger people with MAFLD may have a longer disease duration, a lower health literacy, and unhealthier lifestyles, which predisposes them to a greater CMM risk (21). Females were more prone to experiencing hepatic fibrosis than males, despite the relatively lower prevalence of NAFLD (41). Additionally, both hepatic nuclear receptors and estrogen play the critical roles in regulating liver lipid metabolic pathways, yet these nuclear receptors were found to be dysregulated in patients with fatty liver disease, which may diminish the anti-inflammation and anti-oxidative effect of estrogen (42). Beyond expectation that the risk of CMM was relative higher in current non-smoker than in current smoker, we speculated this difference may be explained by the higher rates of hypertension, hyperlipidemia, insulin resistant, and metabolic syndrome in smokers, which might mask the influence of MAFLD on the CMM to some extent (43). However, whether this association was a coincidence was needed confirmed in further studies.

Although biological mechanisms underlying the association between MAFLD and CMM remains poor understood, several pathways may be the major contributors. First, MAFLD may be the hepatic manifestation of metabolic syndrome, a pathological condition characterized by several metabolic dysfunctions. A large number of studies have documented the stronger associations of metabolic syndrome and individual CMDs (44). Second, inflammation and oxidative stress may two important contributors linking the MAFLD and CMM incidence. MAFLD is associated with the overexpression of pro-inflammation cytokines and the higher circulating levels of inflammation and oxidative stress factors (such as C-reactive protein, oxidized-LDL, plasma plasminogen activator inhibinor-1, and soluble NOX2-derived peptide), all of which can promote the development of atherosclerosis and CVD (45). Third, hepatic steatosis in MAFLD can damage mitochondrial function and hepatic peroxisomes, causing the reduced release of fibroblast growth factor 21 (Fgf21). Fgf21 is an important cardio-protected hepatokines in regulating the energy expenditure, glycemic control, and cardiovascular function, and its treatment promote lowers the blood pressure and protect heart against the oxidative stress (46, 47). Fetuin-A, another hepatokines, is found increased in MAFLD patients and may promote the insulin resistance to induce the development of T2D and CVDs (45).

To our knowledge, our study is the first to investigate the role of MAFLD in the CMM progression, and our results not only confirmed the clinical significance of MAFLD, but provided critical evidence for the early prevention of CMM. The major strengths of this study included the prospective cohort design, a large sample size, the longer follow-up period, the two-stage analytic strategy, and the application of multistate models. However, several limitations should be noted when interpreting our results. First, due to the unavailable data of liver imaging or biopsy in UK Biobank, we used the FLI to define the hepatic steatosis. However, guides recommended the utility of FLD to define the MAFLD and its accuracy was validated previously (15, 48). Second, we only used the single assessment of MAFLD at baseline, which may not comprehensively capture the metabolic characteristics of MAFLD patients. Future studies with longitudinally repeated measurements are needed to investigate the dynamic metabolic changes in MAFLD and its association with CMDs and CMM. Third, since the diagnosis of disease incidence relied on multiple sources, diagnostic date may delay, which may influence the observed disease trajectories. Forth, due to the UK Biobank is not a nationally representative study, our findings are not appropriate to generalize to other populations. Finally, there may exist several unmeasured residual confounders underlying the association between MAFLD and CMM, owing to the nature of observational study design.

## 5 Conclusions

In conclusion, this population-based cohort study revealed the detrimental influences of MAFLD on the whole CMM progression, including most transitions from baseline to individual CMDs, and subsequently to CMM. Given the higher prevalence and severe consequences of CMM, our findings suggest that developing and implementing the effective treatment measures for MAFLD may have profound implications for the primary prevention of CMM.

## Data Availability

The datasets presented in this study can be found in online repositories. The names of the repository/repositories and accession number(s) can be found below: https://www.ukbiobank.ac.uk.
