# Supplementary material for: Association between metabolic-associated fatty liver disease and risk of cardiometabolic multimorbidity: a disease trajectory analysis in UK Biobank
Source: Front Endocrinol (Lausanne). 2025 Jun 18;16:1585725. doi: 10.3389/fendo.2025.1585725 (PMC12213339; doi:10.3389/fendo.2025.1585725)
Supplement: Supplementary file 1 [file DataSheet1.pdf]

Supplementary Table 1 Association between subtypes of metabolic-associated fatty liver disease (MAFLD) and cardiometabolic multimorbidity

	n	Cases/person-years	Unadjusted	Model 1	Model 2	Model 3
Individuals free of cardiometabolic disease at baseline						
Non-MAFLD group	223852	1536/3092787	Ref.	Ref.	Ref.	Ref.
Obese MAFLD group	7509	60/103822	1.16 (0.90-1.50)	1.11 (0.85-1.43)	1.12 (0.86-1.45)	1.12 (0.87-1.45)
Lean MAFLD group	2390	54/32991	3.29 (2.51-4.32)	2.42 (1.84-3.18)	1.99 (1.52-2.62)	1.96 (1.49-2.58)
Obese and metabolic dysfunctional MAFLD group	110664	2972/1520841	3.94 (3.71-4.20)	3.31 (3.10-3.53)	2.96 (2.78-3.16)	2.87 (2.69-3.07)
Individuals with type 2 diabetes at baseline						
Non-MAFLD group	5485	953/69373	Ref.	Ref.	Ref.	Ref.
Obese MAFLD group	37	11/427	1.90 (1.05-3.44)	1.97 (1.09-3.57)	1.99 (1.10-3.61)	2.00 (1.10-3.63)
Lean MAFLD group	144	27/1797	1.10 (0.75-1.61)	0.92 (0.62-1.34)	0.84 (0.57-1.23)	0.82 (0.56-1.20)
Obese and metabolic dysfunctional MAFLD group	13190	3047/162014	1.37 (1.28-1.48)	1.29 (1.20-1.39)	1.23 (1.14-1.33)	1.22 (1.13-1.31)
Individuals with coronary heart disease at baseline						
Non-MAFLD group	8645	984/113698	Ref.	Ref.	Ref.	Ref.
Obese MAFLD group	203	23/2693	0.99 (0.65-1.49)	1.02 (0.68-1.55)	1.05 (0.69-1.59)	1.05 (0.69-1.59)
Lean MAFLD group	176	27/2258	1.39 (0.95-2.04)	1.37 (0.93-2.01)	1.24 (0.85-1.83)	1.23 (0.84-1.81)
Obese and metabolic dysfunctional MAFLD group	9604	2120/119995	2.06 (1.91-2.22)	2.05 (1.90-2.22)	1.94 (1.80-2.10)	1.92

						(1.78-2.08)
Individuals with stroke at baseline						
Non-MAFLD group	2586	431/32925	Ref.	Ref.	Ref.	Ref.
Obese MAFLD group	55	11/671	1.25 (0.69-2.28)	1.22 (0.67-2.22)	1.22 (0.67-2.24)	1.25 (0.69-2.29)
Lean MAFLD group	47	10/566	1.35 (0.72-2.52)	1.26 (0.67-2.36)	1.07 (0.57-2.00)	1.07 (0.57-2.01)
Obese and metabolic dysfunctional MAFLD group	2061	604/24542	1.89 (1.67-2.14)	1.79 (1.58-2.03)	1.68 (1.48-1.91)	1.67 (1.47-1.90)

Abbreviation: MAFLD metabolic-associated fatty liver disease.

Obese MAFLD group included those with hepatic steatosis plus obesity only; lean MAFLD group included those with hepatic steatosis plus metabolic dysfunction only; and obese and dysfunction MAFDL group included those with hepatic steatosis plus obesity and metabolic abnormalities.

Data were presented as hazard ratios (95% confidence intervals).

Model 1 adjusted for age and sex;

Model 2 adjusted for model 1 plus ethnicity, educational levels, family income, socioeconomic status, employed status, smoking status, alcohol drinking, physical activity, sleep duration and healthy diet score;

Model 3 adjusted for model 2 plus family history of diabetes, hypertension, heart disease and stroke.

Supplementary Table 2 Role of subtypes of metabolic-associated fatty liver disease (MAFLD) in the development of cardiometabolic multimorbidity

Exposure	Transitions from baseline status to CMM					
	Baseline to T2D	Baseline to CHD	Baseline to stroke	T2D to CMM	CHD to CMM	Stroke to CMM
Non-MAFLD group	Ref.	Ref.	Ref.	Ref.	Ref.	Ref.
Obese MAFLD group	1.21 (1.01-1.46)	1.01 (0.92-1.10)	1.00 (0.84-1.19)	1.03 (0.55-1.95)	0.88 (0.57-1.37)	1.12 (0.63-2.01)
Lean MAFLD group	2.82 (2.32-3.42)	1.37 (1.21-1.54)	1.13 (0.89-1.43)	1.95 (1.21-3.12)	0.92 (0.56-1.51)	1.47 (0.75-2.87)
Obese and metabolic dysfunctional MAFLD group	5.26 (5.03-5.49)	1.45 (1.41-1.49)	1.12 (1.07-1.18)	1.08 (0.94-1.25)	1.77 (1.60-1.96)	1.68 (1.44-1.96)

Abbreviation: MAFLD metabolic-associated fatty liver disease; T2D type 2 diabetes; CHD coronary heart disease; CMD cardiometabolic disease; CMM cardiometabolic multimorbidity.

Obese MAFLD group included those with hepatic steatosis plus obesity only; lean MAFLD group included those with hepatic steatosis plus metabolic dysfunction only; and obese and dysfunction MAFDL group included those with hepatic steatosis plus obesity and metabolic abnormalities.

Multi-state models were conducted to estimate the role of MAFLD in the development of cardiometabolic multimorbidity, and disease transitions were presented in Figure 2. Data were presented as hazard ratios (95% confidence intervals).

Model adjusted for age, sex, ethnicity, educational levels, family income, socioeconomic status, employed status, smoking status, alcohol drinking, physical activity, sleep duration, healthy diet score, family history of diabetes, hypertension, heart disease and stroke.

Supplementary Table 3 Sensitivity analyses for the association between metabolic-associated fatty liver disease (MAFLD) and cardiometabolic multimorbidity after multiple imputation of missing data on covariates

	Individuals free CMD	Individuals with T2D	Individuals with CHD	Individuals with stroke
Non-MAFLD	Ref.	Ref.	Ref.	Ref.
MAFLD	2.79 (2.62-2.97)	1.21 (1.13-1.31)	1.89 (1.75-2.05)	1.66 (1.46-1.88)
MAFLD with low fibrosis	2.64 (2.47-2.82)	1.17 (1.07-1.28)	1.79 (1.65-1.94)	1.58 (1.38-1.80)
MAFLD with mild fibrosis	3.76 (3.41-4.16)	1.21 (1.12-1.31)	2.23 (2.00-2.48)	2.15 (1.74-2.66)
MAFLD with severe fibrosis	4.03 (1.67-9.71)	1.55 (1.31-1.83)	5.34 (3.08-9.24)	

Abbreviation: MAFLD metabolic-associated fatty liver disease; T2D type 2 diabetes; CHD coronary heart disease.

Data were presented as hazard ratios (95% confidence intervals).

Model adjusted for age, sex, ethnicity, educational levels, family income, socioeconomic status, employed status, smoking status, alcohol drinking, physical activity, sleep duration, healthy diet score, family history of diabetes, hypertension, heart disease and stroke.

Supplementary Table 4 Sensitivity analyses for the association between metabolic-associated fatty liver disease (MAFLD) and cardiometabolic multimorbidity after excluding those occurring events within the first 2 years of follow-up

	Individuals free CMD	Individuals with T2D	Individuals with CHD	Individuals with stroke
Non-MAFLD	Ref.	Ref.	Ref.	Ref.
MAFLD	2.77 (2.60-2.95)	1.20 (1.11-1.30)	1.93 (1.78-2.09)	1.71 (1.50-1.97)
MAFLD with low fibrosis	2.62 (2.45-2.81)	1.14 (1.04-1.26)	1.83 (1.67-1.99)	1.62 (1.40-1.87)
MAFLD with mild fibrosis	3.71 (3.36-4.11)	1.20 (1.11-1.31)	2.27 (2.03-2.54)	2.33 (1.87-2.92)
MAFLD with severe fibrosis	4.07 (1.69-9.80)	1.59 (1.33-1.89)	4.56 (2.44-8.52)	

Abbreviation: MAFLD metabolic-associated fatty liver disease; T2D type 2 diabetes; CHD coronary heart disease.

Data were presented as hazard ratios (95% confidence intervals).

Model adjusted for age, sex, ethnicity, educational levels, family income, socioeconomic status, employed status, smoking status, alcohol drinking, physical activity, sleep duration, healthy diet score, family history of diabetes, hypertension, heart disease and stroke.

Supplementary Table 5 Sensitivity analyses for the association between metabolic-associated fatty liver disease (MAFLD) and cardiometabolic multimorbidity considering the competing risk of death from other causes

	Individuals free CMD	Individuals with T2D	Individuals with CHD	Individuals with stroke
Non-MAFLD	Ref.	Ref.	Ref.	Ref.
MAFLD	2.78 (2.60-2.96)	1.21 (1.13-1.31)	1.90 (1.75-2.05)	1.65 (1.45-1.88)
MAFLD with low fibrosis	2.63 (2.46-2.81)	1.17 (1.07-1.29)	1.79 (1.65-1.94)	1.57 (1.37-1.80)
MAFLD with mild fibrosis	3.73 (3.37-4.12)	1.21 (1.12-1.31)	2.23 (2.00-2.48)	2.15 (1.73-2.67)
MAFLD with severe fibrosis	4.01 (1.64-9.80)	1.55 (1.31-1.83)	5.30 (2.80-10.02)	

Abbreviation: MAFLD metabolic-associated fatty liver disease; T2D type 2 diabetes; CHD coronary heart disease.

Data were presented as hazard ratios (95% confidence intervals).

Model adjusted for age, sex, ethnicity, educational levels, family income, socioeconomic status, employed status, smoking status, alcohol drinking, physical activity, sleep duration, healthy diet score, family history of diabetes, hypertension, heart disease and stroke.

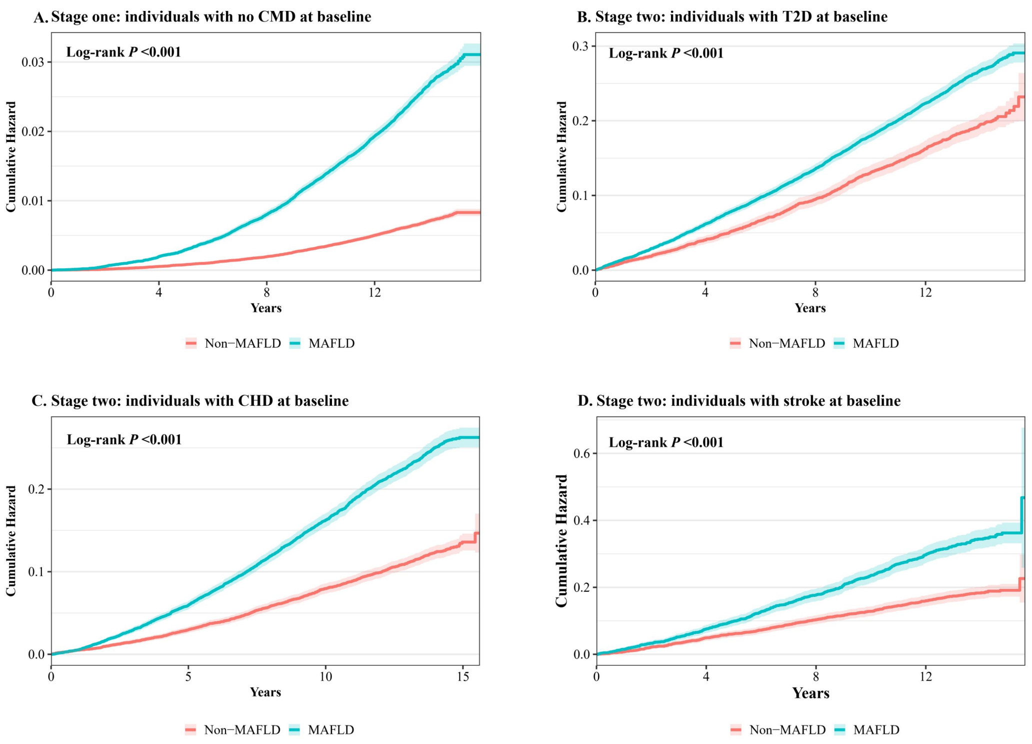

Supplementary Figure 1 The cumulative hazard of incident CMM by MAFLD among individuals with no CMD (A), T2D (B), CHD (C), and stroke (D) at baseline

Abbreviation: CMM cardiometabolic multimorbidity; CMD cardiometabolic disease; T2D type 2 diabetes; CHD coronary heart disease.

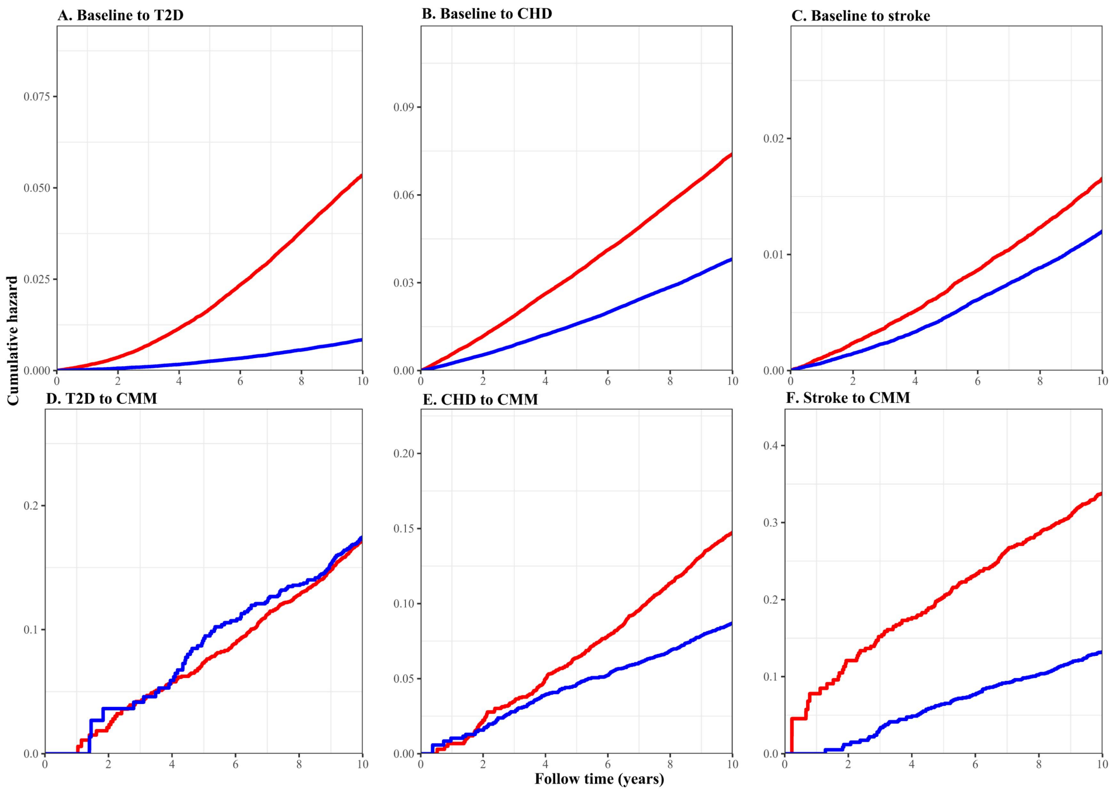

Supplementary Figure 2 Cumulative hazard of different transitions from baseline to individual cardiometabolic disease, and to cardiometabolic multimorbidity stratified by MAFLD

Note: red lines indicated the MAFLD group, and blue lines indicated the no MAFLD group

Subgroup	HR (95% CI)	P for interaction
**Age**		<0.001
<60	3.71 (3.31-4.16)	
>=60	2.40 (2.22-2.59)	
**Gender**		0.001
Female	3.07 (2.79-3.39)	
Male	2.52 (2.32-2.74)	
**Drinking status**		0.793
Not current drinking	2.91 (2.43-3.48)	
Current drinking	2.76 (2.57-2.95)	
**Obesity**		0.801
Yes	2.24 (1.71-2.94)	
No	2.41 (2.23-2.61)	
**Smoking status**		<0.001
Not current smoking	2.94 (2.73-3.15)	
Current smoking	2.30 (1.99-2.66)	
**Socioeconomic status**		0.278
Low	2.85 (2.62-3.10)	
Middle or high	2.66 (2.41-2.94)	
**Physical activity**		0.576
Low	2.74 (2.41-3.13)	
Moderate	2.96 (2.58-3.41)	
High	2.68 (2.37-3.04)	

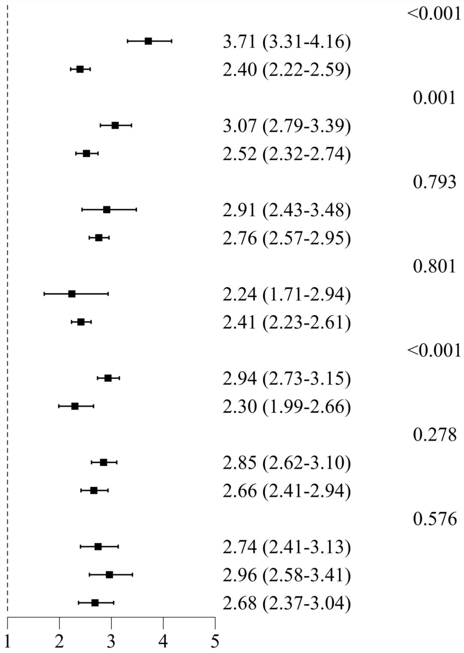

Supplementary Figure 3 Subgroup analyses for the association between metabolic-associated fatty liver (MAFLD) and cardiometabolic multimorbidity among individuals free of cardiometabolic multimorbidity

Note: Model adjusted for age, sex, ethnicity, educational levels, family income, socioeconomic status, employed status, smoking status, alcohol drinking, physical activity, sleep duration, healthy diet score, family history of diabetes, hypertension, heart disease and stroke.

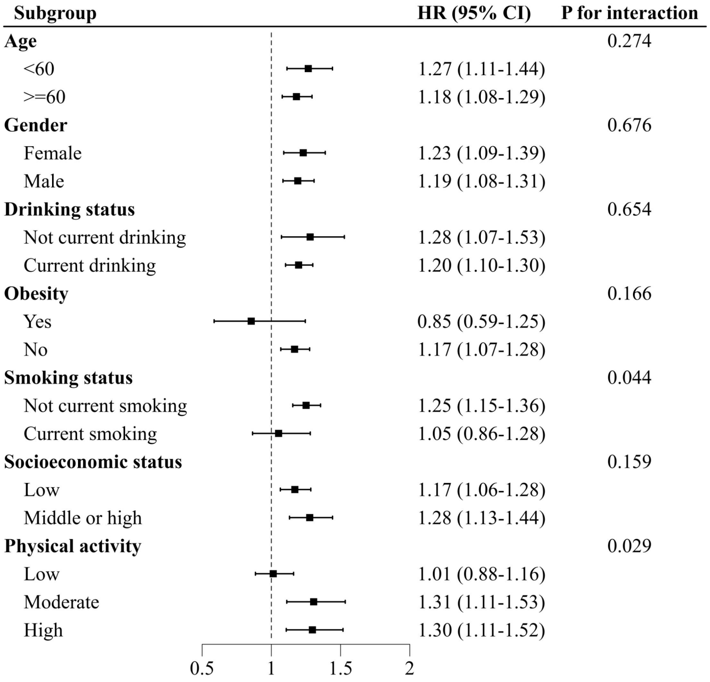

Supplementary Figure 4 Subgroup analyses for the association between metabolic-associated fatty liver (MAFLD) and cardiometabolic multimorbidity among individuals with type 2 diabetes at baseline

Note: Model adjusted for age, sex, ethnicity, educational levels, family income, socioeconomic status, employed status, smoking status, alcohol drinking, physical activity, sleep duration, healthy diet score, family history of diabetes, hypertension, heart disease and stroke.

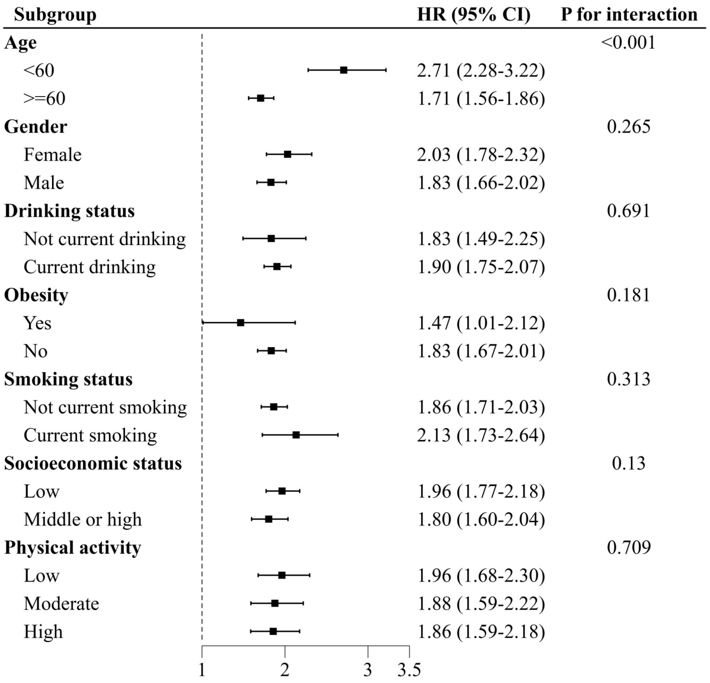

Supplementary Figure 5 Subgroup analyses for the association between metabolic-associated fatty liver (MAFLD) and cardiometabolic multimorbidity among individuals with coronary heart disease at baseline

Note: Model adjusted for age, sex, ethnicity, educational levels, family income, socioeconomic status, employed status, smoking status, alcohol drinking, physical activity, sleep duration, healthy diet score, family history of diabetes, hypertension, heart disease and stroke.

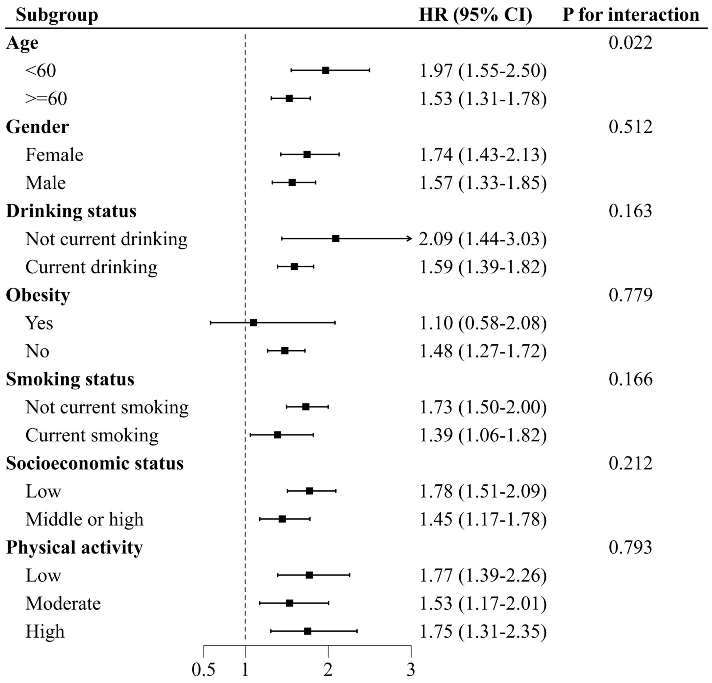

Supplementary Figure 6 Subgroup analyses for the association between metabolic-associated fatty liver (MAFLD) and cardiometabolic multimorbidity among individuals with stroke at baseline

Note: Model adjusted for age, sex, ethnicity, educational levels, family income, socioeconomic status, employed status, smoking status, alcohol drinking, physical activity, sleep duration, healthy diet score, family history of diabetes, hypertension, heart disease and stroke.
